# Hydrogen‐Stabilized Self‐Rectifying Memristor Arrays for Reliable Multilevel Synapses in Transformer‐Based Keyword Spotting

**DOI:** 10.1002/advs.76640

**Published:** 2026-07-20

**Authors:** Seonjeong Lee, Seohyeon Ju, Won Joo Lee, Myounggon Kang, Sungjun Kim, Yoon Kim

**Affiliations:** ^1^ Department of Intelligent Semiconductor Engineering University of Seoul Seoul Republic of Korea; ^2^ Division of Electronics and Electrical Engineering Dongguk University Seoul Republic of Korea; ^3^ Department of Electrical and Computer Engineering University of Seoul Seoul Republic of Korea; ^4^ Department of Intelligent Semiconductor Engineering School of Advanced Fusion Studies University of Seoul Seoul Republic of Korea

**Keywords:** hydrogen annealing, incremental step pulse with verify algorithm, keyword spotting, resistive random‐access memory, self‐rectifying behavior

## Abstract

This study proposes a strategy to simultaneously improve conductance uniformity and data retention characteristics by introducing the incremental step pulse with verify algorithm (ISPVA) technique and hydrogen (H_2_) annealing into a non‐filamentary TiN/Ti/HfO_2_/TiO_x_/TiN resistive switching memory device. The high Schottky barrier formed at the Ti/HfO_2_ interface induces asymmetric electron injection and limits reverse current flow, resulting in a rectifying ratio of approximately 1442. This self‐rectifying characteristic provides an intrinsic advantage in suppressing sneak currents in crossbar arrays. The ISPVA technique improves the linearity and uniformity of conductance modulation, enabling the implementation of up to 6‐bit multilevel states within a few‐µA current range. In addition, H_2_ annealing stabilized conduction by forming hydrogen bonds with oxygen vacancies in the oxide layer and suppressing oxygen ion–vacancy recombination. As a result, data retention over 10^4^ s and endurance exceeding 10^4^ cycles were achieved even under a low energy consumption of 36.3 pJ. Furthermore, the experimentally obtained long‐term potentiation and depression characteristics were implemented in a Transformer‐based keyword spotting (KWS) model, achieving a recognition accuracy of 92.5%. These results suggest that the proposed device enables controlled analog conductance modulation with improved stability, showing its potential for Transformer‐based neuromorphic computing applications.

## Graphical abstract

A non‐filamentary self‐rectifying HfO_2_/TiO_x_ resistive random‐access memory integrates H_2_ annealing with an incremental step pulse with verify algorithm to achieve stable and linear analog conductance modulation. The device enables low‐current 6‐bit multilevel operation while suppressing sneak current in crossbar arrays. Implemented in Transformer‐based keyword spotting, it achieves high recognition accuracy, highlighting its potential for reliable neuromorphic edge intelligence.

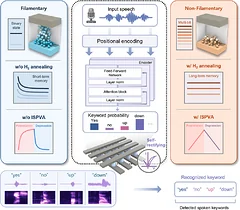

## 1 Introduction

Recent advances in artificial intelligence (AI), particularly in Transformer‐based large language models, have increased the demand for new computing architectures capable of supporting both parallel computation and low‐power data processing [1, 2, 3]. In particular, keyword spotting (KWS) has been widely used for wake‐word recognition in various voice‐based interfaces, including smartphones, wearable devices, smart home appliances, and automotive platforms [4, 5]. These KWS models operate based on self‐attention mechanisms after converting mel‐spectrograms into temporal patches, requiring frequent memory access during repetitive computation processes [6]. Conventional von Neumann architectures suffer from structural limitations in achieving high performance and low‐power computation because the processing unit and memory are physically separated, leading to bottlenecks during large‐scale neural network operations [7, 8]. As an alternative approach, neuromorphic computing, which mimics the structure and information processing mechanisms of the human brain, has attracted considerable attention [9, 10, 11, 12]. Neuromorphic systems perform weight storage and computation simultaneously within synaptic devices, thereby reducing data transfer between memory and processing units and enabling highly efficient and low‐power operation. To realize such systems, various memory devices have been proposed, including phase‐change memory, ferroelectric memory, magnetoresistive memory, and resistive random‐access memory (RRAM) [13, 14, 15, 16]. Among these candidates, RRAM has attracted particular interest as a neuromorphic synaptic device owing to its simple structure, low operating voltage, and fast switching speed. In particular, as illustrated in Figure 1a, the RRAM crossbar array structure enables a minimum cell area of 4F^2^, allowing high integration density and the parallel execution of large‐scale operations such as vector–matrix multiplication (VMM) [17]. These characteristics are suitable for hardware implementation of the self‐attention operation repeatedly performed in Transformer‐based KWS models [18, 19, 20]. In self‐attention, VMM between the query, key, and value weights and the input features is a core operation, requiring repeated multiplication and accumulation processes. In an RRAM crossbar array, the trained query, key, and value weights can be mapped to the conductance states of individual cells, while the input features are applied as voltages [21, 22]. This allows multiplication and accumulation between the input voltage and conductance to be performed in parallel within the array [23, 24]. However, this crossbar‐based parallel computing structure becomes increasingly affected by nonideal current paths and device‐to‐device variations as the array size increases. In particular, as shown in Figure 1b, sneak currents can occur through unselected cells in crossbar structures without cell transistors, and accumulated cell‐to‐cell variations can degrade the read margin and computing accuracy. Although inserting a two‐terminal selector in series with the RRAM cell can suppress sneak currents, it introduces additional process complexity and increases device variability [25]. To mitigate these limitations, RRAM devices with self‐rectifying characteristics and the incremental step pulse with verify algorithm (ISPVA) technique have been proposed [26, 27, 28, 29]. Self‐rectifying RRAM suppresses reverse current by asymmetrically controlling electron injection through the Schottky barrier formed at the metal–oxide interface. The ISPVA technique gradually increases the programming voltage and verifies the resistance at each step, thereby improving the convergence accuracy toward the target resistance and mitigating switching variability. These approaches have mainly been applied to filamentary RRAM devices with excellent long‐term retention characteristics to enhance weight precision [30, 31, 32]. However, filamentary RRAM requires relatively high operating currents because its switching behavior relies on the formation and rupture of conductive filaments. In contrast, non‐filamentary RRAM typically operates through energy barrier modulation associated with charge trapping and detrapping, enabling low‐current operation. Nevertheless, its data retention characteristics can be limited by volatile behavior depending on trap stability [33, 34, 35]. Recently, studies have reported that oxygen (O_2_) and hydrogen (H_2_) annealing processes can regulate defect states by reducing trap density and suppressing ion–vacancy recombination [36, 37, 38]. These approaches have mainly been investigated in high‐k dielectric‐based devices and oxide semiconductor thin‐film transistors, and even when applied to RRAM, they have primarily focused on stabilizing filamentary switching paths. In addition, existing non‐filamentary RRAM‐based neuromorphic systems have mainly been applied to long short‐term memory (LSTM)‐based computing architectures for temporal information processing [39, 40]. However, self‐attention operations in Transformer‐based KWS models simultaneously require high weight precision, stable multilevel conductance states, and low energy consumption. Therefore, an operational strategy combining self‐rectifying behavior, ISPVA‐based conductance modulation, and H_2_ annealing‐based defect‐state stabilization is required. In this work, we propose a non‐filamentary TiN/Ti/HfO_2_/TiO_x_/TiN RRAM device that integrates self‐rectifying behavior, ISPVA programming, and H_2_ annealing within a single device structure. Rather than independently applying these techniques, the proposed approach establishes a coupled operation condition in which reverse current suppression, gradual conductance modulation, and trap‐state modification simultaneously contribute to device operation. As a result, stable conductance states can be maintained with reduced energy consumption while mitigating retention degradation. The proposed device exhibits a rectifying ratio (RR) greater than 1442, a read current below 9 µA, stable data retention exceeding 10^4^ s, and endurance over 10^4^ cycles, while enabling 6‐bit multilevel operation through the ISPVA technique. In addition, the switching mechanism was investigated using x‐ray photoelectron spectroscopy (XPS) and secondary ion mass spectrometry (SIMS) analyses. Furthermore, a Transformer‐based KWS system was implemented using experimentally controlled synaptic weights mapped through a signed 7‐bit representation, achieving a recognition accuracy of 92.5%. This study demonstrates a device structure and operation strategy for Transformer‐based neuromorphic systems while addressing variability and retention degradation in non‐filamentary RRAM.

**FIGURE 1:**
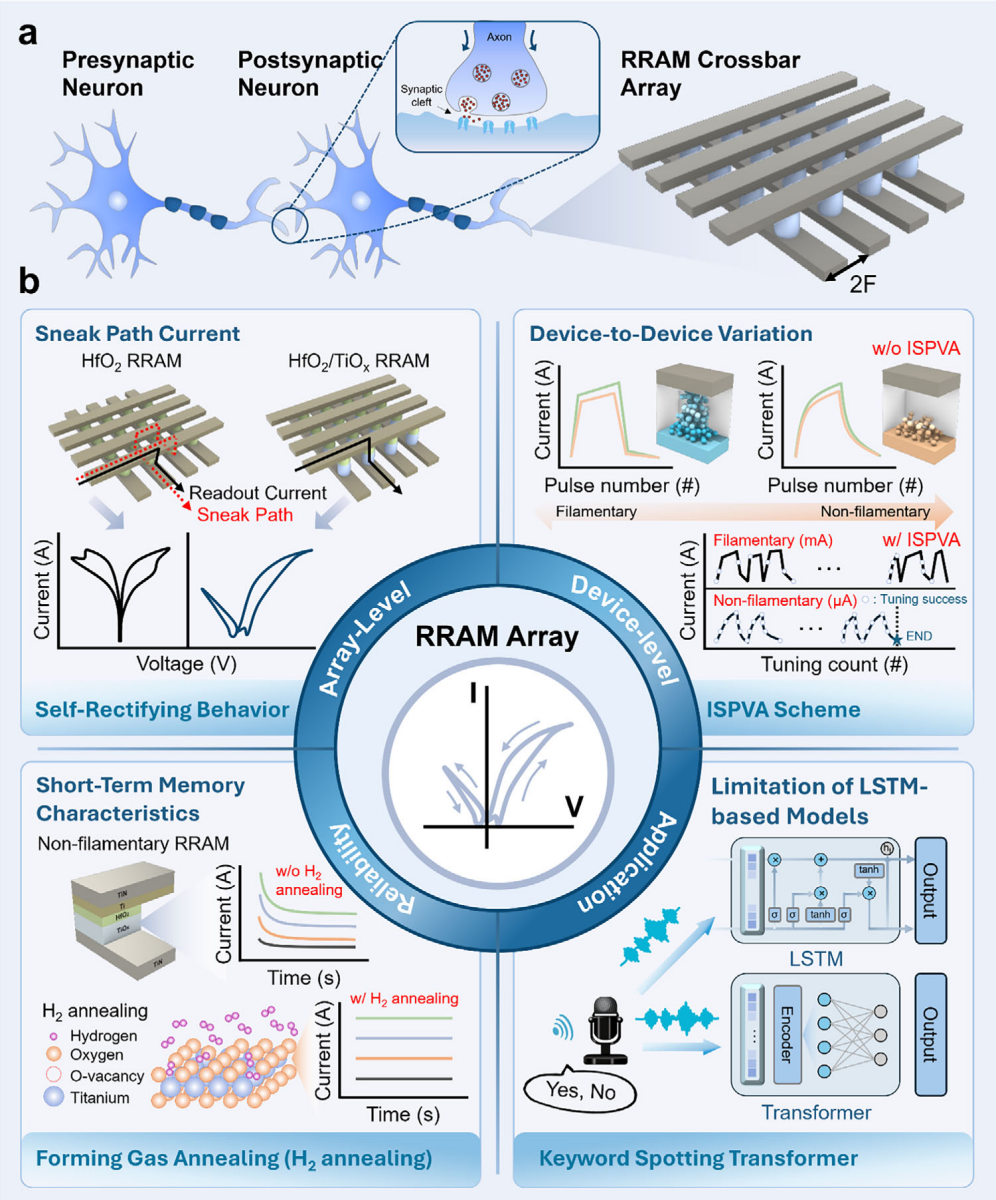
(a) Schematic illustration of an RRAM crossbar array structure mimicking the operating principle of a biological synapse. (b) Conceptual diagram of an RRAM‐based KWS system integrating self‐rectifying characteristics, the ISPVA technique, and the H_2_ annealing process.

## 2 Results and Discussion

Figure 2a,b shows a 3D schematic illustration and a scanning electron microscopy (SEM) image of the TiN/Ti/HfO_2_/TiO_x_/TiN crossbar structure, respectively. A 32 × 32 array was fabricated on a SiO_2_/Si substrate, confirming the realization of an active area of 2.5 × 2.5 µm^2^. Figure 2c presents a cross‐sectional transmission electron microscopy (TEM) image of a single device, where the thicknesses of the HfO_2_ and TiO_x_ layers were measured to be approximately 8 and 20 nm, respectively. The EDS line‐scan was performed along the cross‐sectional direction across the entire device stack, as indicated in the TEM image in Figure S1a. The EDS line‐scan and mapping results shown in Figure S1b,c clearly distinguish the spatial distribution of Ti, N, Hf, and O elements according to the stacked structure. To analyze the chemical composition of the insulating layers, XPS depth profiling was performed while sequentially etching the layers using an argon (Ar) ion beam. Figure 2d,e presents the depth profiles of the Hf 4f and O 1s core levels measured from the upper insulating layer of the HfO_2_/TiO_x_‐based RRAM device after H_2_ annealing. As shown in Figure 2f, the Hf 4f spectrum consists of two peaks corresponding to Hf 4f_7/2_ (16.6 eV) and Hf 4f_5/2_ (18.2 eV), which agree well with the standard Hf^4+^ (HfO_2_) phase [41, 42]. No significant shift in binding energy was observed during the etching process, suggesting that a stoichiometric HfO_2_ layer was formed through the Atomic Layer Deposition (ALD) process. Figure 2g,h shows the Ti 2p and O 1s depth profile results measured from the lower insulating layer. As presented in Figure 2i, the Ti 2p spectrum exhibits Ti 2p_3/2_ and Ti 2p_1/2_ peaks. The peaks corresponding to Ti^4+^ (TiO_2_) were observed at 459.2 and 465.1 eV, while those corresponding to Ti^3+^ (Ti_2_O_3_) appeared at 457.4 and 463.3 eV [43, 44]. The coexistence of Ti^4+^ and Ti^3+^ components indicates the formation of a sub‐oxide state within the TiO_x_ layer. Figure S2a,b present the O 1s depth profile results measured from the HfO_2_ and TiO_x_ layers, respectively. The O 1s spectrum of the HfO_2_ layer was deconvoluted into metal–oxygen (M–O), oxygen vacancy (Vo^2+^), and metal–hydroxyl (M–OH) components, with peaks observed at 530.2, 531.0, and 531.6 eV, respectively. The same three components were also identified in the TiO_x_ layer at 529.7, 531.2, and 532.0 eV, respectively, which are consistent with the binding energy ranges reported in the literature for H_2_‐annealed HfO_2_ and TiO_x_ [45, 46]. Additionally, Figure S3a,b shows cross‐sectional TEM images and the corresponding fast Fourier transform (FFT) analysis results. The FFT analysis performed on the TiO_x_ region revealed diffraction patterns corresponding to anatase TiO_2_ (101) with a d‐spacing of ≈ 0.35 nm and anatase TiO_2_ (004) with a d‐spacing of ≈ 0.23 nm. These diffraction patterns suggest that partial crystallization occurred in the TiO_x_ layer during the Rapid Thermal Annealing (RTA) process, indicating the coexistence of localized crystalline regions within the predominantly amorphous structure [47, 48].

**FIGURE 2:**
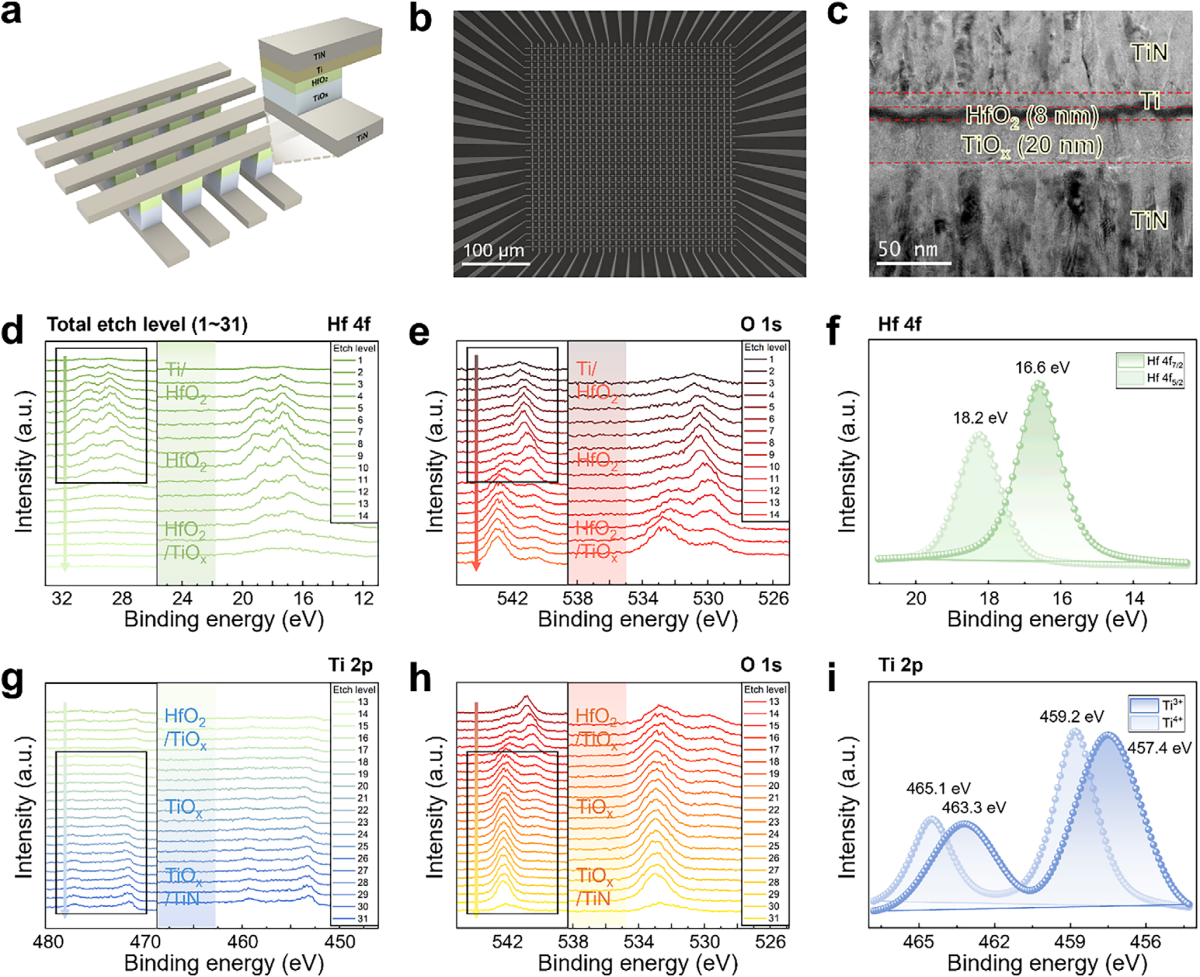
(a) Three‐dimensional schematic illustration of the RRAM crossbar array. (b) Top‐view SEM image of the 32 × 32 array. (c) Cross‐sectional TEM image of the TiN/Ti/HfO_2_/TiO_x_/TiN stacked structure. (d) XPS depth profile of the Hf 4f core level. (e) XPS depth profile of the O 1s core level in the HfO_2_ layer. The left panel shows the raw data, while the right panel presents an enlarged view of the selected region. (f) Peak fitting results of the Hf 4f spectrum in the HfO_2_ layer. (g) XPS depth profile of the Ti 2p core level in the TiO_x_ layer. (h) XPS depth profile of the O 1s core level in the TiO_x_ layer. (i) Peak fitting results of the Ti 2p spectrum in the TiO_x_ layer.

To evaluate the electrical characteristics of the devices depending on the introduction of the TiO_x_ layer and the application of the H_2_ annealing process, the *I*–*V* characteristics were measured by applying a voltage to the top electrode (TE) while grounding the bottom electrode (BE). Figure 3a shows the *I*–*V* characteristics of the HfO_2_‐based RRAM device. As shown in the inset, an electroforming behavior characterized by a sudden increase in current was observed at approximately 2.5 V. The electroforming process was carried out under a positive voltage sweep from 0 to 3 V with a compliance current (I_cc_) of 1 µA. After forming, the device exhibited reversible resistive switching behavior depending on the polarity of the applied voltage. The reset operation, corresponding to the transition from the low‐resistance state (LRS) to the high‐resistance state (HRS), was performed by sweeping a negative voltage from 0 to − 1.2 V at the TE without I_cc_. Conversely, the set operation, corresponding to the transition from HRS to LRS, was performed by sweeping a positive voltage in the range of 0–1.2 V. This switching behavior can be explained by oxygen ion migration. When a positive voltage is applied, O^2−^ ions migrate toward the TE, resulting in the accumulation of Vo^2+^ near the BE. The accumulated Vo^2+^ induce the formation of conductive filaments, switching the device from HRS to LRS. In contrast, when a negative voltage is applied, O^2−^ ions migrate toward the BE and recombine with Vo^2+^, causing the rupture of the conductive filaments and returning the device from LRS to HRS [49, 50]. Figure 3b,c shows the *I*–*V* characteristics of the HfO_2_/TiO_x_‐based RRAM devices depending on whether the H_2_ annealing process was applied. Both devices exhibited electroforming‐free switching behavior without an initial electroforming process, and stable resistive switching characteristics were observed under voltage sweeps. The set operation was performed by sweeping a positive voltage from 0 to 4 V at the TE without I_cc_, while the reset operation was performed by sweeping a negative voltage from 0 to − 2 V under the same conditions. Unlike the filament‐dominated switching observed in the single‐layer HfO_2_ device, the stacked structure exhibited non‐filamentary switching behavior based on charge trapping and detrapping without abrupt formation of conductive filaments. In particular, the HfO_2_/TiO_x_‐based RRAM device showed self‐rectifying characteristics in which the current was suppressed in the reset region compared with the HfO_2_‐based RRAM device. This behavior is attributed to the asymmetric Schottky barriers formed at the Ti/HfO_2_ and TiO_x_/TiN interfaces, which limit electron injection under negative voltage conditions. These self‐rectifying and nonlinear *I*–*V* characteristics were quantified by the rectifying ratio (RR) and nonlinearity (NL) values. Here, RR was defined as |I(+V)|/|I(−V)| at the corresponding read or low‐bias voltage, while NL was defined as |I(V_read_)|/|I(V_read_/2)|, representing the suppression of half‐bias current relative to the selected read current. The increased RR indicates effective suppression of reverse‐bias current, whereas NL indicates suppression of low‐bias leakage current in the stacked oxide structure. Because sneak currents in selector‐free crossbar arrays can flow through unselected or partially selected cells under reverse‐bias or half‐bias conditions, both RR and NL are important parameters for evaluating read reliability [51]. The array‐level effect of these parameters is further quantified by the read‐margin analysis in Figure S10. To analyze the conduction mechanisms, linear fitting was performed using Schottky emission, Poole‐Frenkel (P‐F), direct tunneling, and Fowler‐Nordheim (F‐N) tunneling models for both the LRS and HRS states, and the results are presented in Figure S4 [52, 53]. The analysis indicates that in the low‐voltage region of the set process, Poole–Frenkel conduction and direct tunneling jointly contribute to the current transport, whereas in the high‐voltage region, Fowler–Nordheim tunneling and Schottky emission become dominant. In particular, Schottky emission was identified as the dominant conduction mechanism in the high‐voltage region of the reset process. These results support that the asymmetric Schottky barriers formed at the interfaces effectively control the conduction behavior. Figure 3d shows the retention characteristics of the HfO_2_‐based RRAM device. The retention measurement for the single‐layer HfO_2_‐based RRAM device was performed under a read voltage of 0.5 V. The device maintained two distinct resistance states within a current range of approximately 60 µA for 10^3^ s, demonstrating nonvolatile behavior. Figure 3e,f compares the retention characteristics of the HfO_2_/TiO_x_‐based RRAM devices with and without H_2_ annealing. The retention measurement for the stacked HfO_2_/TiO_x_ device was performed under a read voltage of 1.25 V. The device without H_2_ annealing exhibited up to four distinguishable resistance levels within a current range of approximately 3 µA; however, the current gradually decreased over time, indicating volatile behavior. In contrast, the device with H_2_ annealing maintained up to eight resistance levels within a current range of approximately 7 µA for 10^3^ s, demonstrating stable nonvolatile characteristics. To evaluate device‐to‐device uniformity after a retention delay, the conductance states were first programmed using the ISPVA technique with a uniform current margin of ∼0.3 µA between adjacent states. After a delay time of ∆t = 500 s, the read currents were measured to analyze the current distributions. As shown in Figure 3g, the single‐layer HfO_2_‐based RRAM device maintained a current difference of approximately 56 µA and exhibited relatively consistent current distribution characteristics after ∆t = 500 s. Figure 3h,i compare the current distributions of ten HfO_2_/TiO_x_‐based RRAM devices with and without H_2_ annealing after Δt = 500 s. The device without H_2_ annealing initially showed four resistance levels within a current range of approximately 4 µA; however, a gradual decrease in current was observed over time. In contrast, the device with H_2_ annealing maintained eight resistance levels with a minimum current difference greater than 0.3 µA, showing relatively clear separation between states. This behavior can be attributed to the formation of M─OH bonds during the H_2_ annealing process, which suppress the release of trapped electrons in the switching layer. As a result, the current degradation over time is mitigated, leading to simultaneous improvement in long‐term retention characteristics and resistance‐level separation [54, 55, 56].

**FIGURE 3:**
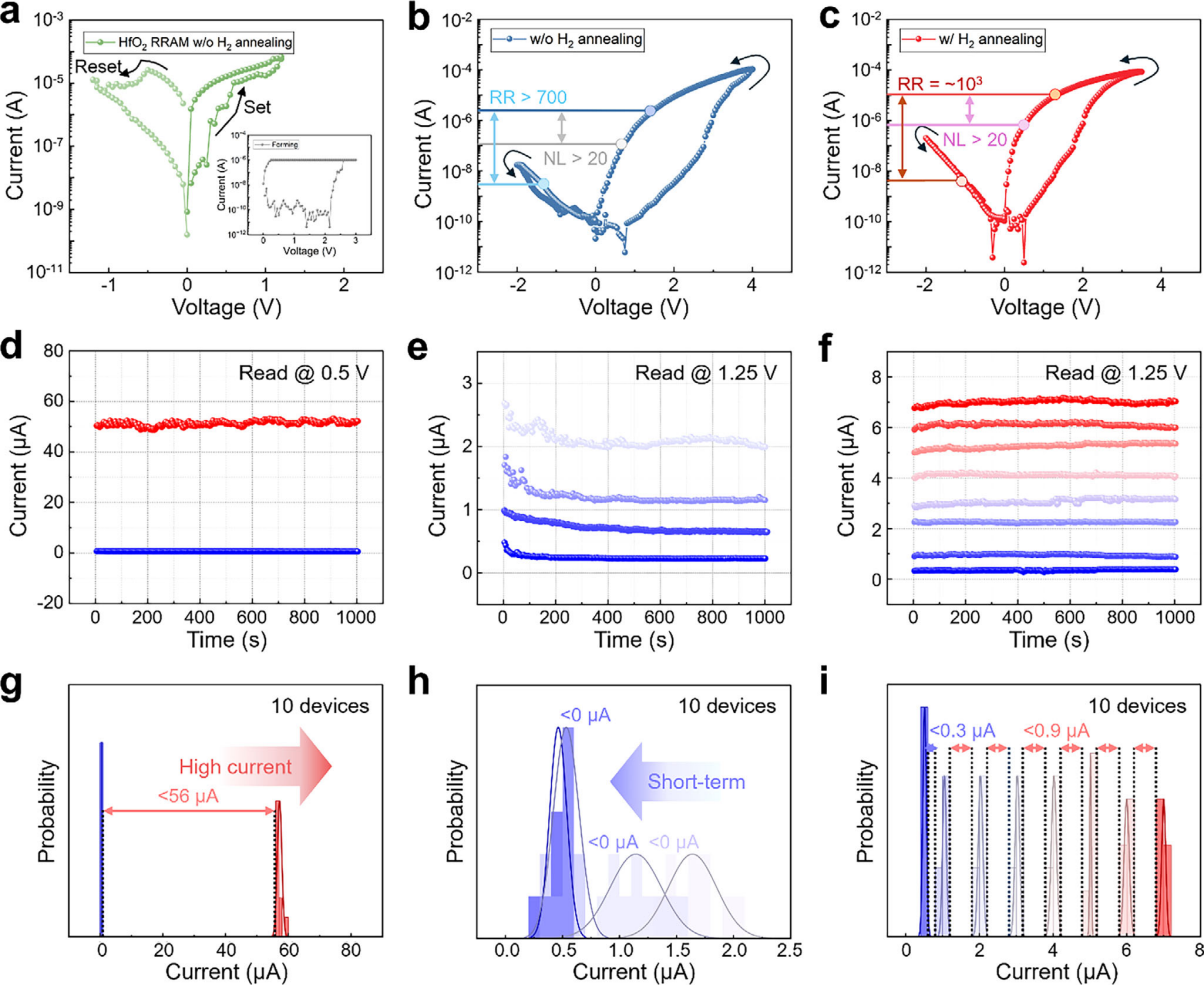
(a) *I*–*V* characteristics of the HfO_2_‐based RRAM device. (b) *I*–*V* characteristics of the non‐annealed TiO_x_/HfO_2_‐based RRAM device. (c) *I*–*V* characteristics of the H_2_‐annealed TiO_x_/HfO_2_‐based RRAM device. (d) Retention characteristics of the HfO_2_‐based RRAM device. (e) Retention characteristics of the non‐annealed TiO_x_/HfO_2_‐based RRAM device. (f) Retention characteristics of the H_2_‐annealed TiO_x_/HfO_2_‐based RRAM device. (g) Conductance distribution of the HfO_2_‐based RRAM device after Δt = 500 s. (h) Conductance distribution of the non‐annealed TiO_x_/HfO_2_‐based RRAM device after Δt = 500 s. (i) Conductance distribution of the H_2_‐annealed TiO_x_/HfO_2_‐based RRAM device after Δt = 500 s.

To investigate the switching mechanism of the TiN/Ti/HfO_2_/TiO_x_/TiN device depending on the presence or absence of H_2_ annealing, Time‐of‐Flight SIMS was performed in the pristine state. Figure 4a shows the negative SIMS depth profile of the HfO_2_/TiO_x_‐based RRAM device with H_2_ annealing. Ti^−^, Hf^−^, O^−^, H^−^, and CsH^−^ ions were analyzed, while the nitrogen component was excluded because negative ion signal formation is limited in the negative mode. The hydrogen distribution was interpreted based on the H^−^ and CsH^−^ signals, where CsH^−^ denotes a cluster ion formed by the combination of cesium (Cs), injected during the sputtering process, with hydrogen [57]. In the device subjected to H_2_ annealing, relatively higher intensities of both H^−^ and CsH^−^ signals were observed in the HfO_2_/TiO_x_ switching layer, particularly near the TiO_x_ region. In addition, as shown in Figure 4b, a comparison of the H^−^ intensity along the depth direction for devices with and without H_2_ annealing revealed that the value of ΔH^−^ = Intensity (H^−^, with H_2_ annealing)—Intensity (H^−^, without H_2_ annealing) was positive in the TiO_x_ layer region. This indicates that the hydrogen concentration increased in this region after annealing, suggesting that the H_2_ annealing performed after TE formation affected the region containing oxygen vacancies in the switching layer. Additionally, as shown in Figure S5a,b, O 1s core‐level XPS depth profile analysis was performed on the non‐annealed HfO_2_/TiO_x_‐based RRAM device, while the same analysis was conducted on the H_2_‐annealed HfO_2_/TiO_x_‐based RRAM device, as shown in Figure S5c,d, in the pristine state. The analysis was performed at the HfO_2_/TiO_x_ interface and TiO_x_ bulk regions, respectively, and M–O, Vo^2+^, and M–OH components were detected in both devices. In the non‐annealed HfO_2_/TiO_x_‐based RRAM device, the Vo^2+^ peak area ratios at the HfO_2_/TiO_x_ interface and TiO_x_ bulk regions were 51.6% and 41.6%, respectively, whereas those of the H_2_‐annealed HfO_2_/TiO_x_‐based RRAM device decreased to 10.6% and 2.5%, respectively. In both devices, a higher Vo^2+^ peak area ratio was observed at the HfO_2_/TiO_x_ interface region compared with the TiO_x_ bulk region, indicating that oxygen vacancy‐related defects were relatively concentrated near the interface region. In addition, the H_2_‐annealed HfO_2_/TiO_x_‐based RRAM device exhibited an overall decrease in the Vo^2+^ component and an increase in the M–OH component, particularly in the TiO_x_ bulk region. These results suggest that H_2_ annealing promotes hydrogen bonding with oxygen vacancies, thereby passivating bulk trap states and stabilizing defect states within the switching layer [38]. As shown in Figure S6, temperature‐dependent measurements revealed that the HRS conduction activation energy increased from 26 to 96 meV after H_2_ annealing, indicating a larger thermally activated transport barrier and supporting the suppression of shallow defect‐mediated leakage paths associated with V_O_‐related transport [58, 59]. Based on these analysis results, the detailed switching mechanisms of the HfO_2_/TiO_x_ devices with and without H_2_ annealing are illustrated in Figure S7. In the device without H_2_ annealing, switching behavior operates based on the generation and recombination of oxygen vacancies, whereas in the H_2_‐annealed device, an additional defect stabilization effect induced by hydrogen bonding with Vo^2+^ is considered to contribute to the switching behavior. This defect stabilization effect reduces the frequency of hard breakdown events and improves device‐to‐device uniformity, as shown in Figure S8a,b. Figure 4c illustrates the retention degradation mechanism in the LRS with and without H_2_ annealing. In the device without H_2_ annealing, Vo^2+^ in the TiO_x_ layer recombine with adjacent O^2−^ ions via thermally activated processes in the LRS. Because Vo^2+^ act as conductive defects, their recombination leads to a reduction in the number of defects participating in conduction. This recombination process also reduces charge trapping states, further destabilizing the conductive path. In contrast, in the device with H_2_ annealing, hydrogen bonds with Vo^2+^ in the TiO_x_ layer, stabilizing the defects. The Vo^2+^–H bonding reduces the mobility and recombination probability of Vo^2+^, thereby suppressing recombination with O^2−^ ions. As a result, Vo^2+^ contributing to conduction are preserved, maintaining stable LRS conductance over time [36].

**FIGURE 4:**
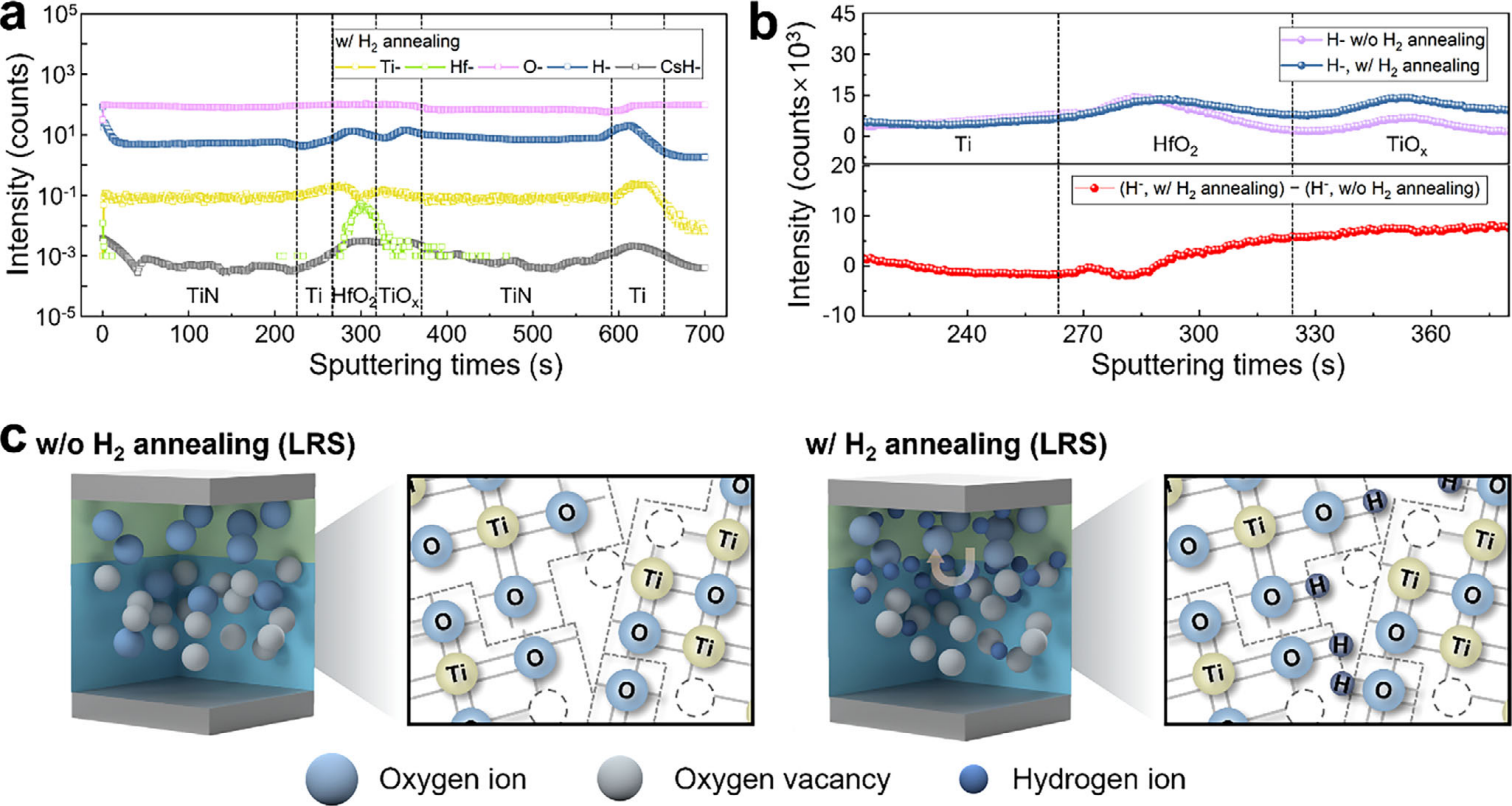
(a) ToF‐SIMS depth profile of the H_2_‐annealed HfO_2_/TiO_x_‐based RRAM device. (b) Comparison of ToF‐SIMS depth profiles of HfO_2_/TiO_x_‐based RRAM devices with and without H_2_ annealing. (c) Schematic illustration of the data retention degradation mechanism in the LRS depending on the presence of H_2_ annealing.

To verify the applicability of the proposed device as a neuromorphic synaptic element, the analog conductance programmability, controllability, and reliability were systematically investigated using different programming schemes. Figure 5a shows the analog *I*–*V* characteristics obtained after applying set voltages in the range of 3.4–4.1 V with a step of 0.1 V, followed by a fixed reset voltage of − 2 V. The device operated without an electroforming process, and the pristine state corresponded to the HRS. The gradual conductance increase with increasing set voltage indicates that the analog states are governed by charge trapping, confirming that the device enables field‐controlled conductance modulation. Figure 5b shows the retention characteristics of the device in the LRS and HRS states. The 3‐bit multilevel current states were maintained for 10^4^ s at room temperature, indicating that not only the binary resistance states but also the intermediate current levels remained distinguishable stably. To further estimate the retention stability beyond 10^4^ s, accelerated retention measurements were performed at 200°C, 220°C, and 240°C, as shown in inset of Figure 5c. The retention failure time, *t*_fail_, was defined as the time at which the current ratio between the LRS and HRS decreased below 10. From the Arrhenius fitting of t_fail_ as a function of 1000/*T* (Figure 5c) [60], activation energy for retention failure was 1.41 eV and the extrapolated retention lifetime at 85°C was 3.84 × 10^8^ s, corresponding to approximately 12.2 years. Figure 5d shows the retention characteristics of 64 conductance levels measured for 10^3^ s at a read voltage of 1.25 V. The 64 states remained distinguishable over the measured retention time, demonstrating the feasibility of 6‐bit conductance modulation. Figure 5e presents the distribution of the 6‐bit target states (G1–G64) implemented using the ISPVA method, where the conductance levels were programmed with clearly separated target‐state distributions. These results suggest that the proposed device is applicable to 6‐bit weight quantization and programming in a 32 × 32 array configuration. Figure 5f presents the AC endurance characteristics measured using the ISPVA method. The set pulses were sequentially applied from 0 to 3 V with a step of 0.1 V, while the reset pulses were applied from 0 to −1 V with a step of −0.01 V. Between each pulse, 1.25 V verify and read pulses were applied, and the pulse width for all operations was set to 100 µs. As a result, the H_2_‐annealed HfO_2_/TiO_x_ device exhibited stable resistive switching endurance over 10^4^ switching cycles under pulse operation while maintaining an On/Off ratio of approximately 10 under the programmed conditions. Figure 5g shows the probability density distributions of the initial state, HRS, and LRS currents extracted from 100 cells at a read voltage of 1.25 V. The coefficient of variation (CV) values of the H_2_‐annealed HfO_2_/TiO_x_ device were 4.39%, 1.99%, and 4.03% for the initial state, HRS, and LRS states, respectively, indicating low variation characteristics below 5% for all states. In addition, Figure S9 presents 10‐cycle DC *I*–*V* curves, including the first cycle, measured from 20 randomly selected cells among the 100 cells. The initial state and HRS exhibited similar average current levels, and despite the relatively larger variation observed in the initial state, electroforming‐free switching characteristics were observed at the same operation voltage. These electroforming‐free characteristics are believed to contribute to the stable cell‐to‐cell uniformity of the H_2_‐annealed HfO_2_/TiO_x_ device, which can be attributed to the relatively uniform distribution and migration behavior of oxygen vacancies involved in the switching process. Moreover, the H_2_‐annealed HfO_2_/TiO_x_ device exhibited relatively narrow current distributions, with sigma values of approximately 0.15 and 0.22 for the HRS and LRS states, respectively. The H_2_‐annealed HfO_2_/TiO_x_ device exhibited higher cell‐to‐cell uniformity compared with the non‐annealed HfO_2_/TiO_x_ device, which can be attributed to the controlled distribution of oxygen vacancies. Figure 5h presents the probability density distributions of the On/Off ratio (I_LRS_(V)/I_HRS_(V)) and the rectifying current ratio (I_LRS_(V)/I_LRS_(− V)) calculated from ten consecutive DC *I*–*V* sweeps. The average On/Off ratio and RR ratio measured from 100 cells reached approximately 148 and 1442, respectively. These 100 cell statistics provide experimental support for the self‐rectifying behavior and reverse‐bias leakage suppression at the unit‐cell level [26]. To estimate how these measured characteristics affect array‐level read reliability, a read margin (RM) analysis was performed based on the LRS/HRS resistance values extracted from the measured *I*–*V* characteristics. Figure S10a shows a schematic diagram of the crossbar array structure used for the RM analysis and the location of the selected cell. When reading a selected cell in a crossbar array, sneak currents flowing through neighboring LRS cells can form alternative current paths, disturbing the output voltage and causing read errors in the HRS state. Figure S10b illustrates the equivalent circuit model based on pull‐up sensing used for the analysis, which includes a resistance network representing the selected cell and the sneak current paths. Under the worst‐case condition, the selected cell was assumed to be in the HRS, while all other cells were assumed to be in the LRS state. The RM is defined based on the difference in output voltage depending on the state of the selected cell and is used as a metric to evaluate the maximum array size that can operate within an acceptable read error range [61]. In this study, RM was defined as $\mathrm{RM}\;\left(N\right)=|V_{\mathrm{out}}^{\mathrm{LRS}}\left(N\right)-V_{\mathrm{out}}^{\mathrm{HRS}}\left(N\right)|/V_{\mathrm{pull}-\mathrm{up}}$ under the condition of V_R_ = 1.25 V [62, 63]. Figure S10c compares the array‐size dependence of RM (N) for the V/2, V/3, and rectifying half‐bias schemes. The voltage distribution applied to the word lines (WLs) and bit lines (BLs) for each bias scheme is presented in Figure S10d. By defining the practical array size as the maximum crossbar line number N that satisfies RM = 10% under the worst‐case condition, the maximum N values for the V/2, V/3, and rectifying half‐bias schemes were calculated to be 21, 335, and 325, respectively. These results suggest that the experimentally measured self‐rectifying characteristics can improve array‐level reliability by mitigating sneak‐current‐induced read disturbance, as estimated by the RM analysis.

**FIGURE 5:**
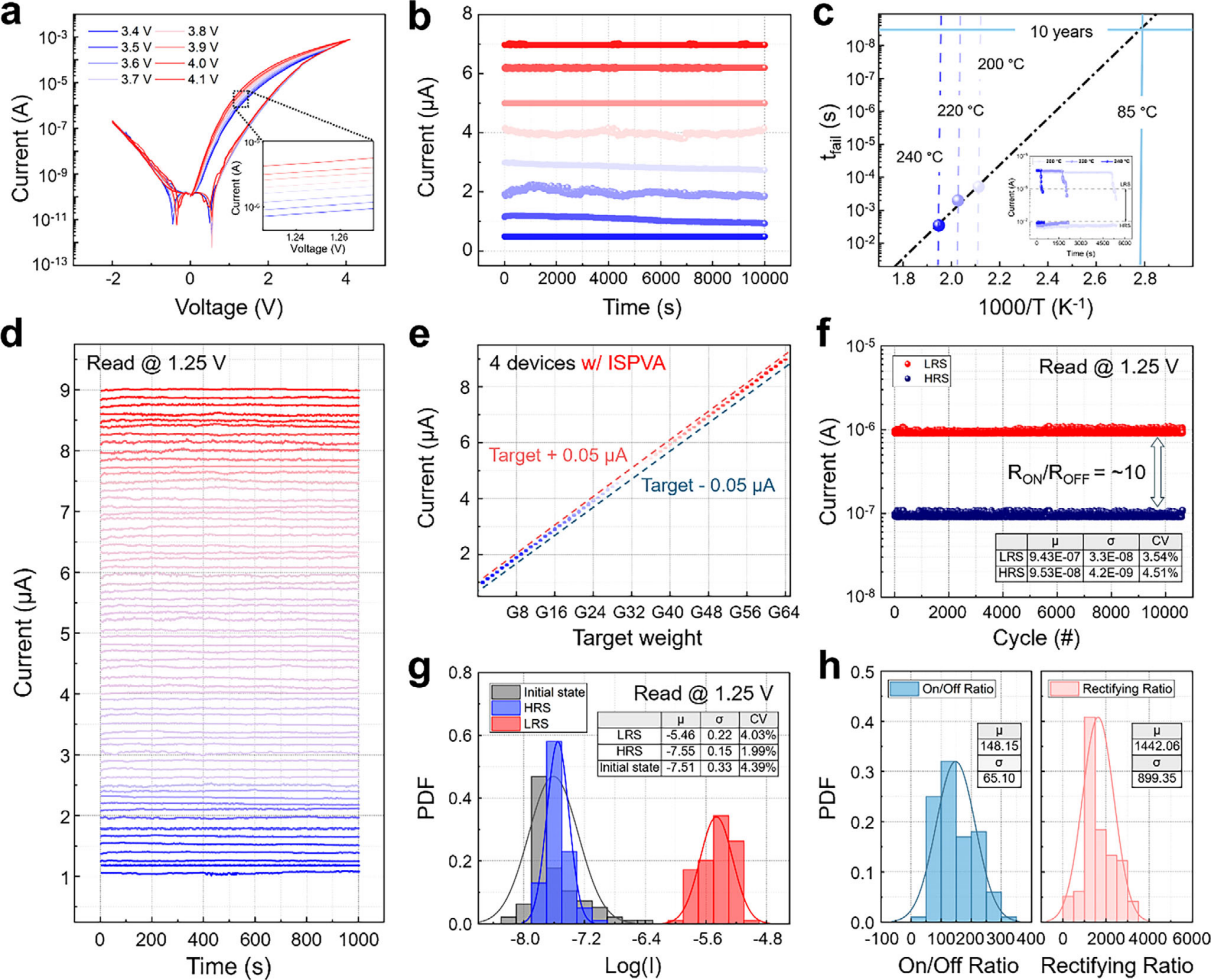
(a) *I*–*V* curves measured under compliance current‐free conditions with different positive voltage sweeps (3.4–4.1 V) and a fixed negative voltage sweep (− 2 V). (b) Retention characteristics of the 3‐bit multilevel states measured for 10^4^ s at a read voltage of 1.25 V. (c) Arrhenius plot of retention failure time, showing the extrapolated data retention characteristics under elevated temperature conditions. (d) Retention characteristics of 64 conductance levels measured for 10^3^ s at a read voltage of 1.25 V. (e) Distribution of the 6‐bit target states (G1–G64) implemented using the ISPVA method. (f) Endurance characteristics of the LRS and HRS repeatedly programmed using the ISPVA method. (g) Current distribution of the LRS, HRS, and initial state measured at a read voltage of 1.25 V. (h) Distributions of the On/Off ratio and rectifying ratio extracted from the H_2_‐annealed HfO_2_/TiO_x_‐based RRAM devices.

To evaluate the system‐level applicability of the proposed RRAM synaptic array for neuromorphic inference, a hardware‐aware KWS Transformer model was implemented [64]. The overall evaluation framework is presented in Figure 6, and the detailed model configuration is summarized in Table 1. As shown in Figure 6a, the KWS model is based on a lightweight encoder‐only Transformer architecture. The overall process consists of three stages: (i) speech preprocessing, (ii) construction and training of the Transformer‐based model, and (iii) incorporation of device characteristics through differential conductance mapping of the trained weights. In the preprocessing stage, the input speech waveform is first converted into a mel‐spectrogram and then divided into 98 non‐overlapping temporal patches. Each patch is projected into a 64‐dimensional embedding space through linear projection, and a learnable class token is prepended. As a result, the final encoder input is represented as a token matrix $X\varepsilon R^{\left(99\times 64\right)}$. The detailed parameters of the mel‐spectrogram and patch configuration are summarized in Table S1. Next, in the KWS model construction stage, the model consists of 12 layers with a model dimension of d_model_ = 64 and a single attention head (d_k_ = 64), as summarized in Table 1. In each layer, the query, key, and value matrices are computed as Q  = XW^Q^ , K  = XW^K^ , and V  = XW^V^ respectively, and the scaled dot‐product attention is defined as shown in Equation (1) [21].

(1) $$\begin{array}{c} Attention\;\left(Q,K,V\right)=Softmax\left(\frac{QK^{T}}{\sqrt{d_{k}}}\right)V \end{array}$$

**FIGURE 6:**
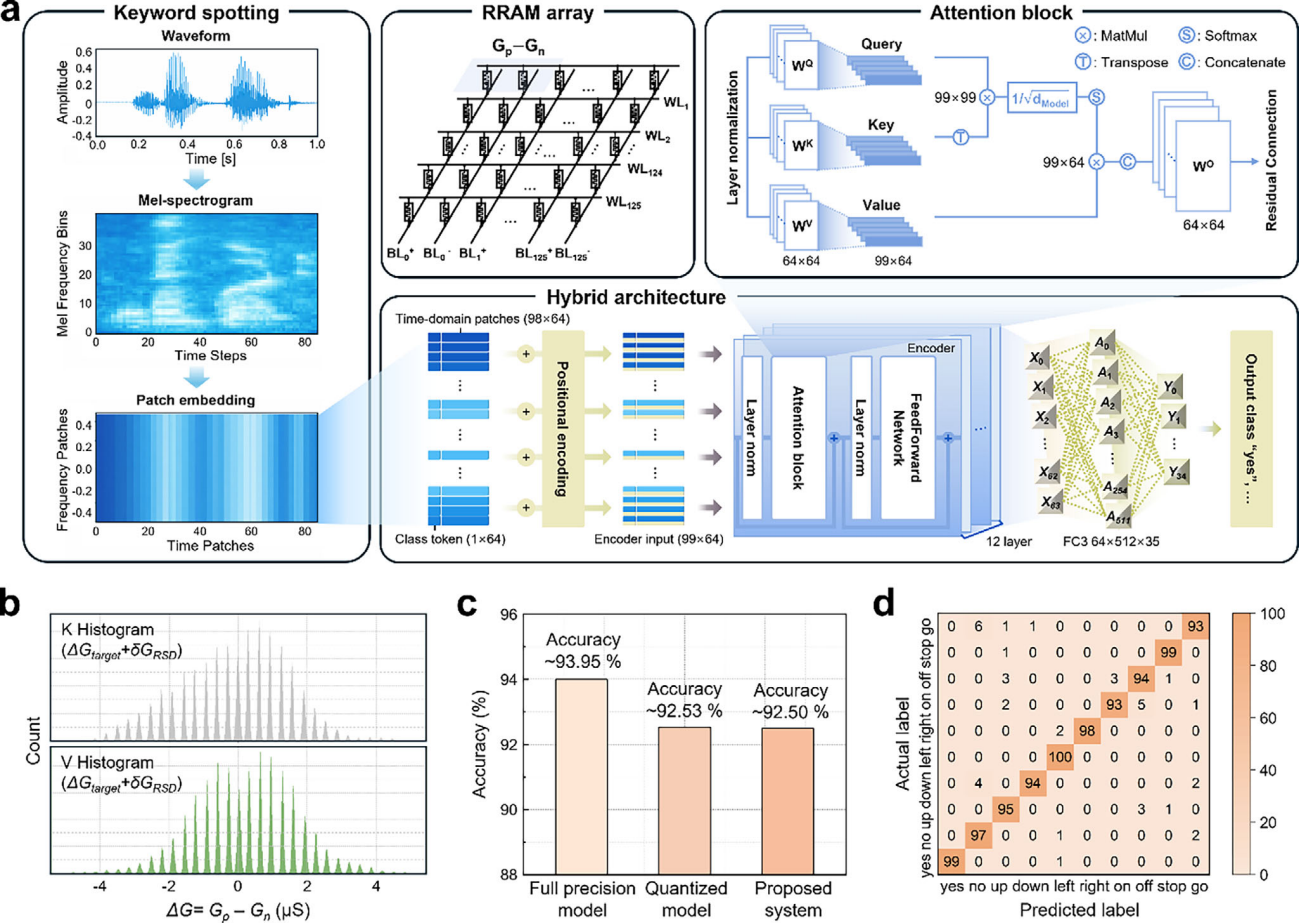
(a) Hybrid architecture including speech waveform preprocessing, a Transformer encoder, and RRAM array‐based computation. The measured conductance values and device variability are mapped to the key and value weights. (b) Distributions of key and value weights incorporating experimentally measured device variability. (c) Comparison of KWS inference accuracy. (d) Class confusion matrix derived from the inference results of the Transformer‐based KWS model considering device variability.

**TABLE 1:** Parameters of the Transformer‐based KWS model.

keyword spotting Transformer Parameter	Value
Model dimension (*d*_model_)	64
Number of heads (*d*_h_)	1
dimension per head (*d*_k_)	64
Numder of Encoder layer	12
Sequance Length of encoder input	99
FeedForward network	64 – 256 – 64
MLP Classifier network	64 – 256 – #classes (35)

After passing Through the 12 encoder layers, the class‐token representation is transformed into the final keyword prediction Through an MLP classifier (64–512–35). The model training was first performed Under full‐precision conditions to establish the baseline performance. In addition, to ensure stable operation Under low‐bit precision and device variability conditions, a loss function combining label smoothing and focal modulation was employed. The detailed configurations of the loss function and training settings are provided in Tables S2 and S3. Finally, for hardware‐aware evaluation, the trained weights were quantized into a signed 7‐bit representation based on a differential conductance structure. First, a 6‐bit magnitude value Within the range of |w_q_| ∈ {0, 1, …, 63}was obtained using the ISPVA technique. Each weight was then mapped to a conductance pair (G_p_, G_n_), where the effective conductance is defined as G =  G_p_ − G_n_. In the array implementation, the positive branch G_p_was mapped Onto ${\mathrm{BL}}_{i}^{+}$, while the negative branch G_n_was mapped onto the adjacent ${\mathrm{BL}}_{i}^{-}$, forming differential conductance pairs. Positive weights were represented as G_p_ =  f(|w_q_|) and G_n_ = G_inactive_ , whereas negative weights were represented as G_n_ =  f(|w_q_|) and G_p_ = G_inactive_ . Here, f(·) denotes a function that maps the 6‐bit magnitude value to discrete conductance levels Between LRS and HRS, while the inactive branch is maintained at the HRS conductance to suppress unnecessary current flow. Through this differential structure, the RRAM crossbar can perform signed accumulation operations based on the input voltage and the conductance difference. The output current can be expressed as Equation (2), enabling analog implementation of signed multiply–accumulate operations Without requiring an additional digital subtraction process [65, 66].

(2) $$\begin{array}{c} \;I_{\mathrm{out}}=\sum_{i}V_{i}\left(G_{p,i}-G_{n,i}\right)\; \end{array}$$

To reflect the non‐ideal characteristics of the measured devices, conductance variability was incorporated into both branches of each differential conductance pair. The variability was modeled as multiplicative Gaussian noise independently applied to all G_p_and G_n_devices, and the conductance including variability is expressed as Equation (3) [67].

(3) $$\begin{array}{c} \;\tilde{G_{p,i}}=G_{p,i}\;\left(1+\delta_{p,i}\right),\;\tilde{G_{n,\;i}}=G_{n,i}\;\left(1+\delta_{n,i}\right)\; \end{array}$$

Here, δ_p,i_ and δ_n,i_ ∼ N(0, σ^2^), where σcorresponds to the CV. The CV was extracted from experimentally measured device statistics and is defined as the ratio of the standard deviation to the mean conductance value. Accordingly, the measured relative standard deviation was used as σin the Gaussian noise model. The effective conductance including variability is defined as ${\tilde{G}}_{i}={\tilde{G}}_{p,i}-{\tilde{G}}_{n,i}$. The conductance distribution broadened by variability is presented in Figure 6b, and the resulting system‐level performance variation is shown in Figure 6c. To further address the hardware implementation feasibility, we describe how the Transformer weight matrices are physically mapped onto the crossbar subarrays. Some operational layers of the Transformer model demand weight matrix dimensions exceeding the physical subarray size. For instance, the linear projection layer mapping 99 audio patches into a 64‐dimensional embedding space requires a 99 × 64 weight matrix. When the differential conductance mapping scheme (G_p_​−G_n_​) is applied for signed MAC operations, the effective physical matrix expands to 256 × 64. Following the tiled mapping strategy established in prior spatially distributed neuromorphic architectures such as ISAAC [68] and ReTransformer [18], each 32 × 32 crossbar array is treated as an independent processing element tile. The 256 × 64 matrix is partitioned into 8 tiles along the row dimension and 2 tiles along the column dimension, yielding a total of 16 subarray tiles that perform parallelized MVM operations in a coordinated manner. This demonstrates the feasibility of scalable mapping of Transformer workloads onto physically constrained RRAM crossbar hardware. Simulation results indicate that the FP32 software baseline model achieves an accuracy of approximately 93.95%. After applying signed 7‐bit quantization, the accuracy decreases to approximately 92.53%. Even when the experimentally measured device variability is incorporated into all differential branches, the proposed RRAM‐based inference system maintains an accuracy of 92.5%, indicating that the additional performance degradation is minimal. In addition, as shown in Figure S11, the ISPVA‐applied device exhibited superior conductance linearity and inference accuracy compared with the device without ISPVA, while detailed measurement conditions and additional simulation results are provided in the Supporting Information. The confusion matrix in Figure 6d exhibits strong diagonal dominance across most keyword classes, demonstrating that stable classification behavior is preserved even under variability‐aware inference conditions. These results suggest that the proposed HfO_2_/TiO_x_‐based RRAM synaptic array, combined with signed differential 7‐bit encoding and realistic conductance variability modeling, can reliably support Transformer‐based neuromorphic inference while minimizing system‐level performance degradation.

Table 2 compares the proposed HfO_2_/TiO_x_‐based RRAM synaptic device with previously reported oxide‐based RRAM devices. Many previous studies have reported filamentary RRAM devices that require I_cc_ and an electroforming process, often exhibiting relatively high read currents. In contrast, the non‐filamentary HfO_2_/TiO_x_‐based device in this work operates without I_cc_ and electroforming. The stacked HfO_2_/TiO_x_ structure is expected to mitigate sneak currents owing to its self‐rectifying characteristics, while the combined use of ISPVA and H_2_ annealing enables stable multilevel conductance modulation. The proposed device maintained 3‐bit and 6‐bit multilevel states for over 10^4^ and 10^3^ s, respectively, and exhibited stable switching characteristics over more than 10^4^ repeated cycles. In addition, Arrhenius analysis of the HRS and LRS estimated nonvolatile retention characteristics exceeding 10 years at 85°C. Figure S12a,b analyzes the write energy consumption of the H_2_‐annealed HfO_2_/TiO_x_ RRAM device based on its transient current response. Compared with previously reported RRAM devices summarized in Table S4, the device exhibited a low energy consumption of 36.3 pJ. This result suggests that the device can achieve multilevel conductance modulation with low write energy while supporting 6‐bit operation. Furthermore, unlike previously reported RRAM‐based KWS neuromorphic systems that mainly rely on LSTM‐based computing‐in‐memory (CIM) processor architectures, this work performs Transformer‐based hardware‐aware inference [69, 70]. By incorporating experimentally measured conductance variation, multilevel retention characteristics, and signed 7‐bit weight representation based on differential conductance mapping, the proposed device achieved an inference accuracy of approximately 92.5% under variability‐aware conditions. These results suggest that the self‐rectifying non‐filamentary RRAM device can support Transformer‐based inference based on stable multilevel states and low write energy.

**TABLE 2:** Performance comparison of oxide‐based RRAM devices for neuromorphic applications.

Device	Switching type	I_cc_	Operating bias			Operating current		On/Off ratio	Retention	Multibit	Endurance	RR	AI application & Accuracy	Reference
			Forming	Set	Reset	On	Off							
Pt/HfO_x_/AlO_x_/TiN	Filamentary	O	O	−2.5 V	3 V	<50 µA	<500 nA	>10	>10^4^ s	3‐bit (>10^3^ s)	>10^2^	—	MNIST: 97.7%	[71]
Ag/TiO_2_/Pt	Filamentary	O	2 V	0.7 V	−0.9 V	<500 µA	<1 nA	∼10^6^	>10^4^ s	—	>50	—	—	[72]
Ag/HfO_2_/ITO	Filamentary	X	X	1.5 V	−1.5 V	<5.4 mA	<1.4 mA	>10	>10^3^ s	6 states (>10^3^ s)	>2.5×10^3^	—	—	[73]
Ti/TiO_2_/p++Si	Filamentary	O	−12 V	−8 V	10 V	<3.5 mA	<1.5 mA	—	>10^4^ s	4‐bit (RC)	—	—	MNIST: 85%	[74]
Pt/HfO_2_/Ti	Filamentary	O	X	2 V	−2 V	<70 µA	<100 nA	>7×10^2^	—	—	—	—	Iris dataset: 98%	[75]
Pt/TiO_x_/TiN	Filamentary	O	3.3 V	−2 V	3 V	<90 µA	<30 µA	>3	>10^4^ s	6‐bit (>10^3^ s)	>10^3^	—	MNIST: 96.4%	[31]
TiN/TiO_2_/WO_x_/Pt	Non‐filamentary	O	X	−4.5 V	2 V	<1.0 µA	<43 nA	>10	Volatile	4‐bit (RC)	>10^2^	—	MNIST: 95.4%	[76]
Pt/Ti/HfO_2_/TiO_x_/Pt/Ti	Non‐filamentary	X	X	4.3 V	−2.5 V	<80 nA	<10 nA	—	—	3‐bit	—	—	MNIST: 95.5%	[77]
TiW/Ai_2_O_3_/Ta_2_O_5_/Ta	Non‐filamentary	X	X	2.5 V	−2.5 V	<4.4 nA	<2 nA	∼50	>10^4^ s	6 states (>10^4^ s)	>10^3^	—	—	[78]
Pt/Al/TiO_x_/HfO_x_/Al_2_O_3_/Pt	Non‐filamentary	X	X	3 V	−3 V	<1 µA	<200 nA	∼10	Volatile	4‐bit (RC)	>10^3^	∼10.5	MNIST: 95.5%	[79]
TiN/TiO_x_/HfO_x_/Pt	Non‐filamentary	X	X	−6 V	5 V	<200 nA	<2 nA	∼10^2^	Volatile	7‐bit (RC)	>10^2^	—	MNIST: 97.8%	[80]
Pt/TaO_x_/HfO_2_/TiN	Non‐filamentary	X	O	6 V	−5 V	<400 nA	<40 nA	>10^2^	>2×10^3^	—	>5×10^2^	∼3743	—	[81]
Pt/TaO_x_/TiN	Non‐filamentary	O	X	2 V	−1.3 V	<80 µA	<10 µA	∼10	Volatile	4‐bit (RC)	>10^2^	—	MNIST: 92.4%, Fashion‐MNIST: 86%	[35]
Ag/HfO_x_/FeO_x_/Au	Non‐filamentary	X	X	2 V	−2 V	<1 A	<100 nA	>10^6^	>10^4^ s	6‐bit (>10^2^ s)	>10^4^	∼10^4^	MNIST: 97%	[82]
Pt/Ta_2_O_5_/Nb_2_O_5‐x_/Al_2_O_3‐y_/Ti	Non‐filamentary	X	X	12 V	−10 V	<1 µA	<1 nA	>10^3^	>10^5^ s	3‐bit(>10^3^ s)	>10^5^	∼5.7×10^4^	MNIST: 91%	[83]
TiN/HfO_x_/Pt	Non‐filamentary	X	X	2 V	−2 V	<2.5 nA	<0.25 nA	∼10	Volatile	—	>10^7^	∼10^8^	Autonomous‐driving dataset: 84.25%	[84]
TiN/Ti/HfO_2_/TiO_x_/TiN/Ti	Non‐filamentary	X	X	3.5 V	−2 V	<9 µA	<800 nA	∼10	>10^4^ s	6‐bit (>10^3^ s), signed 7‐bit	>10^4^	∼1442	Keyword spotting (transformer): 92.5%	This work

## 3 Conclusion

In this study, we propose a TiN/Ti/HfO_2_/TiO_x_/TiN RRAM device that simultaneously exhibits electroforming‐free operation, low‐current operation, self‐rectifying characteristics, and nonlinear behavior by combining the ISPVA technique with an H_2_ annealing process. The proposed device enables stable analog conductance modulation with excellent cell‐to‐cell uniformity. By applying ISPVA to the self‐rectifying RRAM array, the linearity of conductance modulation was improved within a current range below 9 µA, enabling the realization of multilevel states up to 6‐bit precision. In addition, the H_2_ annealing process enabled stable retention of both high‐resistance and low‐resistance states for more than 10^4^ s, stable switching operation over 10^4^ endurance cycles, and reduced device‐to‐device variability within the array. Furthermore, a Transformer‐based KWS model was implemented using the experimentally measured device characteristics. When devices incorporating ISPVA and H_2_ annealing were used, the recognition accuracy was maintained at approximately 92.5% even under long‐term operation conditions. These results demonstrate the potential of the proposed RRAM system for energy‐efficient edge neuromorphic hardware applications.

## 4 Experimental Section

The fabrication of the TiN/Ti/HfO_2_/TiO_x_/TiN device began with the preparation of a thermally oxidized SiO_2_/Si substrate. Organic residues and metallic contaminants on the substrate surface were removed using a standard SPM cleaning process. Subsequently, a 10 nm Ti layer and a 100 nm TiN layer were sequentially deposited by RF reactive sputtering with a Ti target to form BE. The Ti layer was deposited at an RF power of 400 W with Ar gas (60 sccm) under a chamber pressure of 5 mTorr, while the TiN layer was deposited at an RF power of 500 W using a mixed gas of Ar (20 sccm) and N_2_ (3 sccm) under a pressure of 1 mTorr. Next, a 20 nm TiO_x_ layer was deposited by DC sputtering using a Ti target to form the switching layer. The deposition was carried out at a DC power of 130 W with a mixed gas of Ar (30 sccm) and O_2_ (0.7 sccm) under a chamber pressure of 10 mTorr. To enhance the surface oxidation of TiO_x_, post‐treatment was performed using RTA in an O_2_ atmosphere (100 sccm) at 5 mTorr for 5 min. Subsequently, an 8 nm HfO_2_ layer was deposited using ALD at a substrate temperature of 470°C under a chamber pressure of 10 mTorr. Prior to TE formation, photolithography using a negative photoresist was carried out to define array patterns with sizes of 2.5 × 2.5, 5 × 5, 10 × 10, 20 × 20, 40 × 40, and 100 × 100 µm^2^. Afterward, a 10 nm Ti layer and a 100 nm TiN layer were sequentially deposited under the same conditions used for BE by RF reactive sputtering with a Ti target to form TE. The TE patterning was completed through a lift‐off process using acetone. Finally, H_2_ annealing was performed using a mini furnace at 300°C for 30 min in a mixed atmosphere of N_2_ (4 slpm) and H_2_ (1 slpm). The structural and chemical properties of the fabricated devices were analyzed using cross‐sectional TEM and XPS. Electrical characterization was carried out using a probe station equipped with a Keithley 4200‐SCS semiconductor parameter analyzer and a 4225‐PMU pulse measurement unit. DC voltage sweeps and ISPVA measurements were conducted with the bias applied to TE while BE was grounded.

## Author Contributions

**Won Joo Lee**: investigation, formal analysis, Writing – original draft. **Seohyeon Ju**: investigation, formal analysis, writing – original draft. **Myounggon Kang**: investigation, formal analysis, validation. **Seonjeong Lee**: formal analysis, investigation, writing – original draft. **Sungjun Kim**: validation, conceptualization, project administration, funding acquisition, writing – review and editing, supervision, resources. **Yoon Kim**: supervision, resources, project administration, writing – review and editing, validation, funding acquisition, conceptualization.

## Conflicts of Interest

The authors declare no conflicts of interest.

## Supporting information




**Supporting File**: advs76640‐sup‐0001‐SuppMat.docx.

## Data Availability

The data that support the findings of this study are available from the corresponding author upon reasonable request.
