# Supplementary material for: Hydrogen‐Stabilized Self‐Rectifying Memristor Arrays for Reliable Multilevel Synapses in Transformer‐Based Keyword Spotting
Source: Adv Sci (Weinh). 2026 Jul 20;13(56):e76640. doi: 10.1002/advs.76640 (PMC13383159; doi:10.1002/advs.76640)
Supplement: Supplementary file 1 [file ADVS-13-e76640-s001.docx]

Supporting Information

**Hydrogen-Stabilized Self-Rectifying Memristor Arrays for Reliable Multilevel Synapses in Transformer-Based Keyword Spotting**

Seonjeong Lee^1†^, Seohyeon Ju^2†^, Won Joo Lee^3†^, Myounggon Kang^4^, Sungjun Kim^2^*, and Yoon Kim^3^*

^1^Department of Intelligent Semiconductor Engineering, University of Seoul, Seoul 02504, Republic of Korea

^2^Division of Electronics and Electrical Engineering, Dongguk University, Seoul 04620, Republic of Korea

^3^Department of Electrical and Computer Engineering, University of Seoul, Seoul 02504, Republic of Korea

^4^Department of Intelligent Semiconductor Engineering, School of Advanced Fusion Studies, University of Seoul, Seoul 02504, Republic of Korea

E-mail: [yoonkim82@uos.ac.kr](mailto:yoonkim82@uos.ac.kr), [sungjun@dongguk.edu](mailto:sungjun@dongguk.edu)

**Figure S1.** a) Cross-sectional TEM image of the TiN/Ti/HfO_2_/TiO_x_/TiN RRAM device with the indicated EDS line-scan position. b) EDS line-scan analysis showing the elemental distribution of Ti, N, Hf, and O across the device stack. c) EDS elemental mapping images of Ti, N, Hf, and O, confirming the layer structure and compositional distribution.

**Figure S2.** a) Peak fitting results of the O 1s spectrum in the HfO_2_ layer. b) Peak fitting results of the O 1s spectrum in the TiO_x_ layer.

**Figure S3.** a) Cross-sectional TEM image of the TiN/Ti/HfO_2_/TiO_x_/TiN stacked structure and the FFT analysis of the selected region. b) High-resolution TEM (HRTEM) image and FFT analysis confirming the anatase TiO_2_ (101) and anatase TiO_2_ (004) crystal planes.

**Figure S4.** a) Energy band diagram of the TiN/Ti/HfO_2_/TiO_x_/TiN structure with the corresponding work functions and electron affinities. b) I–V characteristics and conduction model fitting results during the set operation. c) I–V characteristics and conduction model fitting results during the reset operation. d) Analysis of the conduction mechanisms in the low- and high-voltage regions during the set operation with the corresponding energy band schematic. e) Analysis of the conduction mechanisms in the low- and high-voltage regions during the reset operation with the corresponding energy band schematic.

To analyze the conduction mechanisms of the HfO_2_/TiO_x_-based RRAM device, the DC I–V characteristics were fitted using Schottky emission, Poole–Frenkel (P–F) emission, direct tunneling, and Fowler–Nordheim (F–N) tunneling models (Figure S4a). Figure S4b,c show the measured I–V curves during the SET and RESET processes, together with the corresponding fitting results for each conduction model. In the low-voltage region of the SET process, the conduction behavior was interpreted by considering Schottky emission, P–F emission, and direct tunneling (Figure S4d(I)). The current densities for Schottky emission and P–F emission are expressed as follows^[S1,S2]^:

$$\begin{aligned} J_{\mathrm{SE}}={4\pi\mathrm{qm}}^{*} \left( \frac{k^{2}T^{2}}{h^{3}} \right)\exp\left[ -\frac{q\left( \phi_{B}-\sqrt{qE/4\pi\varepsilon} \right)}{kT} \right]\#\left( S1 \right) \end{aligned}$$

$$\begin{aligned} J_{\mathrm{PF}}= q\mu N_{C}E exp\left[ -\frac{q\left( \phi_{T}- \sqrt{qE/\pi\varepsilon} \right)}{\mathrm{kT}} \right]\#\left( S2 \right) \end{aligned}$$

where $m^{*}$is the effective electron mass, $h$ is Planck’s constant, $q$is the elementary charge, $k$ is Boltzmann’s constant, $T$ is the absolute temperature, $E$ is the electric field, $\phi_{B}$is the Schottky barrier height, $\phi_{T}$ is the trap energy level, $\varepsilon$ is the dielectric permittivity, $\mu$ is the carrier mobility, and $N_{C}$ is the density of states in the conduction band. Each conduction mechanism can be identified by linearizing its exponential dependence on the electric field. Schottky emission shows a linear relationship in the ln(J)–E^1/2^ plot, whereas P–F emission exhibits linearity in the ln(J/E)–E^1/2^ plot. These linear relationships indicate field-assisted thermal emission, in which the effective barrier height is lowered by the applied electric field. In the low-voltage region of the SET process, electrons are injected under the influence of the Schottky barrier at the TiO_x_/TiN interface, and some electrons can also be injected through P–F emission via trap states near the interface. The injected electrons are then affected by the barrier at the HfO_2_/TiO_x_ interface, which prevents an abrupt increase in current and leads to a gradual current increase. In addition, some electrons can pass through the thinned HfO_2_ barrier via direct tunneling. As shown in Figure S4d(II), F–N tunneling and Schottky emission become the main conduction mechanisms in the high-voltage region of the SET process. The current density for F–N tunneling is given by^[S1–S2]^:

$$\begin{aligned} J_{\mathrm{FN}}= \left( \frac{q^{2}E^{2}}{8\pi h\phi_{B}} \right)\exp\left[ \frac{-8\pi\sqrt{2qm^{*}}\phi_{B}^{\frac{3}{2}}}{3hE} \right]\#\left( S3 \right) \end{aligned}$$

F–N tunneling exhibits a linear relationship in the ln(I/V^2^)–1/V plot, indicating that the barrier becomes sufficiently thin and triangular under a high electric field, allowing electrons to tunnel through the barrier by field emission. Therefore, in the high-voltage SET region, electrons are injected across the HfO₂ barrier under a strong electric field, while Schottky emission at the interfacial barrier also contributes to the increase in current.

In the low-voltage region of the RESET process, Schottky emission, direct tunneling, and P–F emission are observed (Figure S4e(I)). Under negative bias, electrons near the TiO_x_/TiN interface tend to be extracted toward the grounded TiN electrode, leading to electron transport within the switching layer. During this process, the barriers formed at the Ti/HfO_2_ and TiO_x_/TiN interfaces limit electron transport and additional electron injection. However, in this voltage region, some electrons can pass through the thinned HfO_2_ barrier via direct tunneling, while P–F emission through trap states also affects the current flow. The fitting results in Figure S4e(I) indicate that tunneling conduction and trap-assisted conduction occur in the low-voltage RESET region. In the high-voltage region of the RESET process, Schottky emission becomes the dominant conduction mechanism (Figure S4e(II)). This behavior is attributed to the asymmetric Schottky barriers formed at the Ti/HfO_2_ and TiO_x_/TiN interfaces. As the negative bias increases, electron transport becomes more strongly limited by the interfacial barriers, thereby suppressing the reverse current flow. This barrier-limited electron transport is closely related to the self-rectifying characteristics observed in the device.

**Figure S5**. a) Peak fitting result of the O 1s spectrum at the HfO_2_/TiO_x_ interface region in the non-annealed HfO_2_/TiO_x_-based RRAM device. b) Peak fitting result of the O 1s spectrum at the TiO_x_ bulk region in the non-annealed HfO_2_/TiO_x_-based RRAM device. c) Peak fitting result of the O 1s spectrum at the HfO_2_/TiO_x_ interface region in the H_2_-annealed HfO_2_/TiO_x_-based RRAM device. d) Peak fitting result of the O 1s spectrum at the TiO_x_ bulk region in the H_2_-annealed HfO_2_/TiO_x_-based RRAM device.

**Figure S6.** Temperature-dependent current characteristics of the HfO_2_/TiO_x_-based RRAM devices with and without H_2_ annealing.

Temperature-dependent read-current measurements were performed for both the LRS and HRS of the HfO_2_/TiO_x_-based RRAM devices with and without H_2_ annealing. The LRS and HRS currents exhibited distinct temperature-dependent responses, indicating that the carrier transport characteristics depend on the resistance state. In the HRS, where current flow is governed by leakage through localized defect-related states rather than a highly conductive path, the temperature-dependent current behavior can provide information on the thermally activated transport barrier. The HRS current was described using the Arrhenius relation^[S3]^:

$$\begin{aligned} I_{\mathrm{HRS}}= I_{0}\exp\left( -\frac{E_{a}}{kT} \right)\#\left( S4 \right) \end{aligned}$$

where $I_{0}$ is the pre-exponential factor, $E_{a}$ is the effective activation energy for HRS conduction, $k$ is the Boltzmann constant, and $T$ is the absolute temperature. By taking the natural logarithm, the equation can be rewritten as

$$\begin{aligned} {ln(I}_{\mathrm{HRS}})= {ln(I}_{0})-\left( \frac{E_{a}}{k}\cdot\frac{1}{T} \right)\#\left( S5 \right) \end{aligned}$$

Accordingly, the effective activation energy was extracted from the slope of the Arrhenius plot of $ln\left( I_{HRS} \right)$ as a function of 1/ $T$, where the slope corresponds to $-E_{a}/k$. The extracted $E_{a}$ increased from 26 meV for the non-annealed device to 96 meV for the H_2_-annealed device. Larger activation energy indicates that HRS conduction in the H_2_-annealed device requires higher thermal energy for energetic configuration of defect-mediated transport paths, possibly by reducing shallow oxygen vacancy leakage components and forming more stable hydrogen-related defect configurations.^[S4]^

**Figure S7.** a) Schematic illustration showing the distribution changes of oxygen ions and oxygen vacancies in the pristine, LRS, and HRS states of the non-annealed TiN/Ti/HfO_2_/TiO_x_/TiN RRAM device. b) Schematic illustration showing the behavior of oxygen ions and oxygen vacancies with hydrogen bonding in the pristine, LRS, and HRS states of the H_2_-annealed TiN/Ti/HfO_2_/TiO_x_/TiN RRAM device.

Based on the SIMS and XPS analysis results, the switching mechanism of the HfO_2_/TiO_x_ device without H₂ annealing was summarized according to each resistance state, as shown in Figure S7a. In the pristine state, oxygen vacancies exist within the switching layer. As revealed by the XPS analysis described earlier, the HfO_2_ layer exhibited a stoichiometric composition, whereas the TiO_x_ layer showed the presence of Ti^3+^ components, indicating a relatively oxygen-deficient characteristic. This suggests that Vo^2+^ involved in the switching process are mainly distributed in the TiO_x_ layer. After the set operation, when the device reaches the LRS, O^2-^ ions migrate toward the HfO_2_ layer, which has a higher Hf–O bond energy, resulting in an increased Vo^2+^ density within the TiO_x_ layer.^[S5]^ The accumulated Vo^2+^ in the TiO_x_ layer act as deep trap states and capture electrons. This charge trapping redistributes the space-charge within the switching layer and reduces the effective barrier at the interface, thereby increasing the conductivity. After the reset operation, when the device returns to the HRS, O^2-^ ions migrate toward the BE and recombine with Vo^2+^. At the same time, the trapped electrons are released, reducing the trap occupancy and alleviating the space-charge effect. Consequently, the effective interfacial barrier is restored and the conductivity decreases.^[S6]^ In contrast, as shown in Figure S7b, in the device with H_2_ annealing, bonds are formed between Vo^2+^ and hydrogen in the pristine state, thereby modulating the trap density. The set and reset operations follow the switching mechanism described above; however, hydrogen-bonded traps suppress excessive charge accumulation and limit the formation of unintended current paths.^[S7]^

**Figure S8.** a) I–V characteristics and hard breakdown behavior of the non-annealed Ti/HfO_2_/TiO_x_/TiN RRAM device. b) Comparison of the LRS and HRS current distributions of ten devices with and without H_2_ annealing.

**Figure S9.** Ten DC I–V curves measured from 20 randomly selected H_2_-annealed HfO_2_/TiO_x_-based RRAM devices.

**Figure S10.** Read margin (RM) simulation for crossbar arrays using the self-rectifying H₂-annealed HfO₂/TiO_x_ RRAM. a) Region partition around the selected cell (cells sharing WL/BL and remaining (N-1)^2^ cells). b) Equivalent pull-up sensing circuit including lumped sneak-path resistances. c) Read voltage margin as a function of the number of crossbar lines (N) for V/2, V/3, and rectifying half-bias schemes; the dashed line indicates the 10% margin criterion. d) WL/BL biasing schemes used in the RM calculation.

Sneak currents in crossbar arrays can generate readout errors in HRS cells by providing additional current paths through neighboring LRS cells. To quantify the practical array scalability, RM was evaluated using a pull-up sensing configuration (Figure S10b). For a worst-case read condition, the selected cell was assumed to be in the HRS while all remaining cells were set to the LRS. The analysis was performed for three read-operation schemes (V/2, V/3, and rectifying half-bias; Figure S10d), which determine the effective bias (magnitude and polarity) applied to unselected/partially-selected cells during readout. The selected-cell resistances were defined at the read voltage V_R_ = 1.25 V. The effective LRS resistances in sneak paths were determined according to the scheme-dependent biasing of unselected/partially-selected cells (Figure S10d). Based on the equivalent circuit, the overall sneak-path resistance and sensing resistances were calculated, and the normalized read margin with respect to the pull-up voltage (V_pu_) was derived from Kirchhoff’s law as follows^[S8–S10]^:

$$\begin{aligned} R_{\mathrm{sneak}}=\frac{R_{\mathrm{LRS}}^{\mathrm{sneak}}}{N-1}+\frac{R_{\mathrm{LRS}}^{\mathrm{sneak}}}{{(N-1)}^{2}}+\frac{R_{\mathrm{LRS}}^{\mathrm{sneak}}}{N-1},\#\left( S6 \right) \end{aligned}$$

$$\begin{aligned} R_{LRS, sensing}=\frac{R_{\mathrm{LRS}}^{\mathrm{sneak}}\times R_{\mathrm{sneak}}}{R_{\mathrm{LRS}}^{\mathrm{sneak}}+R_{\mathrm{sneak}}}, \#\left( S7 \right) \end{aligned}$$

$$\begin{aligned} R_{HRS, sensing}=\frac{R_{\mathrm{HRS}}^{\mathrm{sneak}}\times R_{\mathrm{sneak}}}{R_{\mathrm{HRS}}^{\mathrm{sneak}}+R_{\mathrm{sneak}}},\#\left( S8 \right) \end{aligned}$$

$$\begin{aligned} \frac{\Delta V}{V_{\mathrm{pu}}}=\frac{V_{out, HRS}}{V_{\mathrm{pu}}}-\frac{V_{out, LRS}}{V_{\mathrm{pu}}}=\frac{R_{\mathrm{pu}}}{R_{HRS, sensing}+R_{\mathrm{pu}}}-\frac{R_{\mathrm{pu}}}{R_{LRS, sensing}+R_{\mathrm{pu}}}\#\left( S9 \right) \end{aligned}$$

$\mathrm{RM}$ was computed as a function of the number of crossbar lines $N$ (Figure S106c). The maximum feasible array size was defined using a worst-case criterion of $\mathrm{RM}$ = 10%, and the corresponding $N_{\max}$values for each scheme were extracted from the $RM(N)$ curves.

**Figure S11.** a) Potentiation and depression characteristics with and without ISPVA. b) Potentiation and depression characteristics according to the 3–6 bit resolution based on ISPVA. c) Comparison of inference accuracy of the Transformer-based KWS model as a function of effective weight bit-width with and without ISPVA.

Figure S11a shows the potentiation and depression characteristics with and without ISPVA. In the case without ISPVA, the set, reset, and read voltages of 3.5 V, −1 V, and 1.25 V were applied for 500 μs, respectively. In contrast, when ISPVA was applied, the pulses were sequentially applied under the same conditions as those used in Figure 5h in the main text. In addition, the target maximum current range was adjusted to 9 μA to implement 64 states in the LTP and LTD curves. To quantitatively compare the potentiation and depression nonlinearities depending on the application of ISPVA, the measured conductance values were fitted using the following equation^[S11]^:

$$\begin{aligned} G=\left\{ \begin{aligned} {[(G_{\mathrm{LRS}}^{\alpha}-G_{\mathrm{HRS}}^{\alpha})\times w+G_{\mathrm{HRS}}^{\alpha}]}^{1/\alpha} if \alpha\neq0 \#\#\#\#\#\# \\ G_{\mathrm{HRS}}\times{(\frac{G_{\mathrm{LRS}}}{G_{\mathrm{HRS}}})}^{w} if \alpha=0 \# \end{aligned} \right.\#\left( S10 \right) \end{aligned}$$

where G_LRS_ and G_HRS_ represent the minimum and maximum conductance values, respectively, and *w* denotes the normalized synaptic weight ranging from 0 to 1. The nonlinearity parameter α quantifies the linearity of conductance modulation during potentiation (α_p_) and depression (α_d_) processes. The fitting results showed that, without ISPVA, the potentiation and depression characteristics exhibited α_p_=4.10 and α_d_=−1.78, respectively, indicating abrupt conductance changes in the initial pulse region and highly nonlinear responses. In contrast, when ISPVA was applied, the potentiation and depression characteristics showed α_p_=1.02 and α_d_=1.00, respectively, while the conductance changed almost linearly within the range of approximately 1–9 μA. These results confirm that ISPVA enables more linear potentiation and depression characteristics. The measured conductance values were mapped as weights for the Transformer-based keyword spotting simulation, and the inference performance was evaluated through software-level simulation. As shown in Figure S11c, the recognition accuracies for the cases with and without ISPVA using signed 7-bit implementation were approximately 92.53% and 92.09%, respectively. These results indicate that the improved conductance linearity achieved by ISPVA contributes to enhanced inference accuracy. Figure S11b presents the potentiation and depression characteristics of the signed 4-, 5-, 6-, and 7-bit states obtained using ISPVA. The experimentally extracted CV values across all conductance levels ranged from 0.09% to 2.93%, and each level was individually assigned its corresponding CV value as the Gaussian noise parameter in the hardware-aware simulation. The corresponding simulation results are summarized in Figure S11c, where the average recognition accuracies were 86.54%, 92.09%, 92.43%, and 92.53%, respectively. As expected, the recognition accuracy increased with increasing bit resolution. In particular, ISPVA provided improved inference accuracy even under low-bit conditions. These results demonstrate that ISPVA-based conductance control enables stable multiple-state implementation and can improve the recognition performance of Transformer-based neuromorphic inference systems.

**Figure S12.** a) Transient current response and energy consumption characteristics of the H_2_-annealed HfO_2_/TiO_x_ RRAM device under the write pulse scheme. b) Transient current characteristics measured in the 18–28 μs region, including the current variation (ΔI) and the switching time (Δt) required to reach the maximum set current level.

**Table S1**. Parameter of Patch embedding for preprocessing of keyword-spotting Transformer

| Patch embedding Parameter | Value |
| --- | --- |
| Window Length | 30 ms |
| Hop Length | 10 ms |
| Number of mel-filter | 40 |
| Mel-spectrogram Dimension | (40, 1) |
| Number of patch | 98 + 1 (class token) |

**Table S2**. Training configuration

| Component | Configuration |
| --- | --- |
| Loss function | Customized cross-entropy with focal and label smoothing terms |
| Optimizer | AdamW |
| Learning rate | Initial value 1×10^-3^, adjusted by scheduler |
| Scheduler | Linear warm-up + cosine annealing |
| Batch size | 512 |
| Epochs | 100 |
| Weight decay | 0.1 |
| Bias | false |

**Table S3**. Data augmentation strategies applied during training

| Augmentation Type | Configuration | Probability |
| --- | --- | --- |
| Time Masking | Up to 20 frames | 70% |
| Frequency Masking | Up to 8 mel bins | 70% |
| Background Noise Injection | Additive noise at 10 dB SNR | 70% |
| Time Shifting | Random shift within ±1600 samples | 70% |
| Temporal Resampling | Scaling factor 0.85×–1.15× | 70% |
| Class Rebalancing | Oversampling factor ×3 for minority classes | Applied to training set |

The input speech signals from the Google Speech Commands v2 dataset were converted into mel-spectrogram representations prior to model training. A 30 ms analysis window with a 10 ms hop length was used, generating 40 mel-frequency bins per frame. The resulting time–frequency representation was segmented into 98 non-overlapping patches of size (40, 1), which were subsequently projected into a 64-dimensional embedding space. The detailed preprocessing parameters are summarized in Table S1. The network was trained in full precision using the AdamW optimizer with an initial learning rate of 1$\times$10^-3^. A linear warm-up phase was first applied, followed by cosine annealing over 100 epochs. A batch size of 512 and a weight decay coefficient of 0.1 were used throughout training. Bias terms were disabled to ensure compatibility with crossbar-based hardware implementation. The complete training configuration, including optimizer and scheduling parameters, is summarized in Table S2. To enhance robustness under quantized inference and device variation, multiple data augmentation techniques were applied during training. Each augmentation was independently applied with a probability of 70%, allowing multiple perturbations to occur simultaneously within a single training sample. The applied augmentations include spectral masking, additive background noise injection at 10 dB signal-to-noise ratio (SNR), temporal shifting within $\pm$1600 samples, and temporal resampling with a scaling factor between 0.85$\times$ and 1.15$\times$. To mitigate class imbalance in the dataset, minority classes were oversampled by a factor of three during training. The detailed augmentation settings are summarized in Table S3. To stabilize training under low-bit precision and hardware-aware inference constraints, a loss function combining label smoothing and focal modulation was adopted. The label-smoothed target distribution is defined as Equation (S11).

$$\begin{aligned} q_{i}=\left( 1-\varepsilon\right)1\left[ i=y \right]+\frac{\varepsilon}{C} \#\left( S11 \right) \end{aligned}$$

where y denotes the ground-truth class index, C is the total number of classes, and ε is the smoothing factor. The term 1[I = y] represents the indicator function, which equals 1 when I = y and 0 otherwise. Accordingly, the ground-truth class is assigned a probability of 1 – ε + ε / C, while all non-ground-truth classes are assigned ε / C. This formulation prevents overconfident predictions and improves robustness under quantized and variation-aware training conditions. The label-smoothed cross-entropy loss is computed as Equation (S12).

$$\begin{aligned} \mathcal{L}_{\text{LS}}=-\sum_{i=1}^{C} q_{i}\log p_{i}\#\left( S12 \right) \end{aligned}$$

Focal modulation is incorporated as Equation (S13),

$$\begin{aligned} \mathcal{L}_{\text{Focal-LS}}=\left( 1-P_{t}^{\text{LS}} \right)^{\gamma}\mathcal{L}_{\text{LS}}\#\left( S13 \right) \end{aligned}$$

where $P_{t}^{\text{LS}}$, defined in Equation (S14), represents the smoothed confidence term,

$$\begin{aligned} P_{t}^{\text{LS}}=\sum_{i=1}^{C} q_{i}p_{i}.\#\left( S14 \right) \end{aligned}$$

Here, γ is the focusing parameter controlling the modulation strength. This training framework establishes the full-precision baseline performance prior to hardware mapping.

**Table S4**. Comparison of the energy consumption characteristics of the H_2_-annealed HfO_2_/TiO_x_ RRAM device with previously reported RRAM studies.

| Devices | Energy consumption | Ref |
| --- | --- | --- |
| Ag/PMMA/3AMP-DJ/PEDOT:PSS/ITO | 26 nJ | [S12] |
| Au/MoS_2_/Au | 200 pJ | [S13] |
| Ag/GeTe/MoTe_2_/Pt | 30 nJ | [S14] |
| TiN/TiO_x_N_y_/SnO_x_/Pt | 3.24 nJ | [S15] |
| Ti/NbO_x_/Pt | 89 pJ | [S16] |
| Graphene/α-ln_2_Se_3_/h-BN/Cr-Au | 75 pJ | [S17] |
| Ag/HfO_x_/C | 40 pJ | [S18] |
| EGaIn/MoS_2_/ITO | 32.9 pJ | [S19] |
| Ti/SnP_2_S_6_/Au | 24 pJ | [S20] |
| TiN/HfO_2_/ITO | 1.74 nJ | [S21] |
| TiN/Ti/HfO_2_/TiO_x_/TiN/Ti | 36.3 pJ | This work |

**References**

S1.	E.W. Lim, and R. Ismail, “Conduction mechanism of valence change resistive switching memory: a survey,” *Electronics* 4 (2015): 586-613, https://doi.org/10.3390/electronics403058.

S2.	M. Ismail, U. Chand, C. Mahata, J. Nebhen, and S. Kim, “Demonstration of synaptic and resistive switching characteristics in W/TiO2/HfO2/TaN memristor crossbar array for bioinspired neuromorphic computing,” *Journal of Materials Science & Technology* 96 (2022): 94-102, https://doi.org/10.1016/j.jmst.2021.04.025.

S3.	H.D. Kim, M.J. Yun, S.M. Hong, H.M. An, and T.G. Kim, “Bias temperature instability analysis on memory properties improved by hydrogen annealing treatment in Ti/HfOx/Pt capacitors,” *physica status solidi (RRL)–Rapid Research Letters* 7 (2013): 497-500, https://doi.org/10.1002/pssr.201307192.

S4.	N. Kopperberg, D.J. Wouters, R. Waser, S. Menzel, and S. Wiefels, "Accurate evaluation method for HRS retention of VCM ReRAM." *APL Materials* 12 (2024), https://doi.org/10.1063/5.0188573.

S5.	K.A. Moltved, and K.P. Kepp, “The chemical bond between transition metals and oxygen: electronegativity, d-orbital effects, and oxophilicity as descriptors of metal–oxygen interactions,” *The Journal of Physical Chemistry C* 123 (2019): 18432-18444, https://doi.org/10.1021/acs.jpcc.9b04317.

S6.	G. Kim, S. Son, H. Song, et al., “Retention secured nonlinear and self‐rectifying analog charge trap memristor for energy‐efficient neuromorphic hardware,” *Advanced Science* 10 (2023): 2205654, https://doi.org/10.1002/advs.202205654.

S7.	J.H. Hong, C.H. Lee, H.W. Kim, et al., “AI-driven quantitative review of mobility–stability trade-off in oxide semiconductors,” *Nano Convergence* 13 (2026): 4, https://doi.org/10.1186/s40580-026-00535-3.

S8.	C.L. Lo, T.H. Hou, M.C. Chen, and J.J. Huang, “Dependence of read margin on pull-up schemes in high-density one selector–one resistor crossbar array,” *IEEE transactions on electron devices* 60 (2012): 420-426, https://doi.org/10.1109/TED.2012.2225147.

S9.	H. So, S. Kim, and S. Kim, “Self-rectifying NiOX/WOX heterojunction synaptic memristor for crossbar architectured reservoir computing system,” *Journal of Alloys and Compounds* 1003 (2024): 175644, https://doi.org/10.1016/j.jallcom.2024.175644.

S10.	Y.C. Chen, C.C Lin, and Y.F. Chang, “Post-Moore memory technology: Sneak path current (SPC) phenomena on RRAM crossbar array and solutions,” *Micromachines* 12 (2021): 50, https://doi.org/10.3390/mi12010050.

S11.	J. Park, M. Kwak, K. Moon, et al., “TiO x-based RRAM synapse with 64-levels of conductance and symmetric conductance change by adopting a hybrid pulse scheme for neuromorphic computing,” *IEEE Electron Device Letters* 37 (2016): 1559-1562, https://doi.org/10.1109/LED.2016.2622716.

S12.	M. Khemnani, B. Tripathi, P. Thakkar, et al., “Investigating the role of interfacial layer for resistive switching in a hybrid Dion-Jacobson perovskite-based memristor,” *ACS Applied Electronic Materials* 5 (2023): 5249-5256, https://doi.org/10.1021/acsaelm.3c01038.

S13.	E. Lee, J. Kim, J. Park, et al., “Realizing electronic synapses by defect engineering in polycrystalline two-dimensional MoS2 for neuromorphic computing,” *ACS Applied Materials & Interfaces* 15 (2023): 15839-15847, https://doi.org/10.1021/acsami.2c21688.

S14.	A.C. Khot, K.A. Nirmal, T.D. Dongale, and T.G. Kim, “GeTe/MoTe2 van der Waals heterostructures: enabling ultralow voltage memristors for nonvolatile memory and neuromorphic computing applications,” *Small* 20 (2024): 2400791, https://doi.org/10.1002/smll.202400791.

S15.	M. Ismail, D. Kim, E. Lim, et al., “Exploration of analog synaptic plasticity and convolutional neural network simulation in bilayer TiO x N y/SnO x memristor for neuromorphic systems,” *ACS Materials Letters* 6 (2024): 3514-3522, https://doi.org/10.1021/acsmaterialslett.4c00406.

S16.	D. Ju, and S. Kim, “Versatile NbOx‐based volatile memristor for artificial intelligent applications,” *Advanced Functional Materials* 34 (2024): 2409436, https://doi.org/10.1002/adfm.202409436.

S17.	X. Liu, C. Zhang, E. Li, et al., “Ultralow off‐state current and multilevel resistance state in van der Waals heterostructure memristors,” *Advanced Functional Materials* 34 (2024): 2309642, https://doi.org/10.1002/adfm.202309642.

S18.	A. Milozzi, S. Ricci, and D. Ielmini, “Memristive tonotopic mapping with volatile resistive switching memory devices,” *Nature Communications* 15 (2024): 2812, https://doi.org/10.1038/s41467-024-47228-1.

S19.	P. Saha, M. Sahad E, S. Sathyanarayana, and B.C. Das, “Solution-processed robust multifunctional memristor of 2D layered material thin film,” *ACS nano* 18 (2023): 1137-1148, https://doi.org/10.1021/acsnano.3c10775.

S20.	T.T.T. Tun, S. Mitra, H. Su, et al., “Intrinsic Nanopore‐Assisted SnP2S6 Memristors With Ti Ion Dynamics for Compact Logic‐In‐Memory Hardware,” *Advanced Functional Materials* (2026): e28751, https://doi.org/10.1002/adfm.202528751.

S21.	Y. Jang, C. Hwang, M. Chae, T. Kim, and H.D. Kim, “Polarity‐Controlled Volatile HfO2 Memristors with Bimodal Conductance for Neuromorphic Synapses and Reservoir Computing,” *Advanced Science* (2026): e15926, https://doi.org/10.1002/advs.202515926.
